# P44 An acutely unwell and rare presentation of systemic lupus erythematosus; a case report

**DOI:** 10.1093/rap/rkae117.075

**Published:** 2024-11-05

**Authors:** Jegadis Sreeneyasan, Kapeel Daive, Easwaradhas Gladston Chelliah, Sarang Chitale

**Affiliations:** Wrightington Wigan & Leigh NHS Foundation trust, Wigan, United Kingdom; Wrightington Wigan & Leigh NHS Foundation trust, Wigan, United Kingdom; Wrightington Wigan & Leigh NHS Foundation trust, Wigan, United Kingdom; Wrightington Wigan & Leigh NHS Foundation trust, Wigan, United Kingdom

## Abstract

**Introduction:**

Systemic lupus erythematosus (SLE) is a systemic autoimmune disease with multisystem involvement. The condition has varying clinical presentations from mild mucocutaneous manifestations to severe multiorgan and central nervous system involvement. It is challenging to diagnose someone with SLE during acute admission. Apart from infection and malignancies, autoimmune and connective tissue diseases should always be one of our differentials in an acutely unwell patient with multisystem involvement. This case demonstrates how unwell and rare a patient with SLE can present. We also acknowledge the need to further understand and raise awareness of this uncommon condition.

**Case description:**

A 48 year old Thai lady with no known medical illness presented with 2-3 weeks history of viral like illness. On admission, she was tachycardic, hypotensive and hypoxic. Clinical examination revealed bi-basal crepitations and elevated JVP. Her ECG showed sinus tachycardia. Admission bloods showed anemia, raised white cell count, lymphopenia, thrombocytopenia, raised inflammatory markers and acute kidney injury (AKI). CT scan revealed evidence of pericardial effusion with small free fluid in abdomen and pelvis. ECHO showed large pericardial effusion and pericardiocentesis was performed which drained purulent fluid. Blood culture was positive for E. Coli. 3rd Day blood tests were strongly positive for CTD screening. She was reviewed by the rheumatology team and was diagnosed as acute presentation of SLE causing serositis having fulfilled the SLICC criteria with E. Coli bacteremia. SLE DAI – 2K score was 11. She reported history of Raynaud’s since moving to UK, hair loss and two miscarriages in the past. She denied history of oral ulcers, facial rash or significant joint involvement. Renal team advised that AKI was due to infection rather than lupus. She was commenced on high dose oral prednisolone (40 mg/day) and antibiotics. Her condition responded to steroids and subsequently was commenced on mycophenolate as outpatient.

**Discussion:**

SLE is an autoimmune disease that affects multiple organ systems. Symptoms and signs can vary although majority of patients present with cutaneous manifestation. Most of the clinical manifestations of the disease are due to abnormal inflammatory response. Patients with SLE are usually diagnosed at outpatient settings although they can present acutely unwell as seen in this patient. Serositis refers to inflammation of serous tissue which includes pleura, pericardium and peritoneum. It is considerably a rare condition which is only seen in only around 10% of patients with SLE. Patients can present with shortness of breath, abdominal pain, diarrhoea, fatigue chest pain and even cardiac tamponade. Due to the heterogeneity of SLE presentations, it is challenging for clinicians to diagnose and manage the symptoms. Besides infection and malignancies, it is always important to consider connective tissue disease as one of the differentials in an acutely unwell patient presenting with multisystem involvement. SLICC criteria can be used as a diagnosing tool for SLE which includes clinical and immunological criteria. SLEDAI-2K score can be used to assess disease activity. Her biochemical parameters as well as clinical symptoms drastically improved following steroid initiation. Early recognition and treatment initiation can improve mortality and morbidity significantly. Despite how unwell she was during admission; she improved quickly and was discharged relatively early. Her condition continues to improve on DMARDs, and she is on regular clinic follow up.

**Key learning points:**

• A good clinical history and examination is essential and aids reaching a diagnosis. As reflected in this case, our patient was a female at childbearing age, south Asian (non-Caucasian ethnicity) who presented with multisystem involvement. Her initial finding of serositis, acute kidney injury, anemia, raised ESR and E. coli bacteremia were not fitting with a typical sepsis picture. There should be strong grounds for considering SLE during such clinical presentations. Suspecting SLE in an acute setting is really challenging due to various reasons, like wide symptomology of SLE, minimal exposure of SLE to physicians and their tendency of leaning towards other common conditions, like sepsis. As SLE is mostly diagnosed as outpatient, inpatient presentations are rare. Furthermore, blood results for connective tissue disease which is a major criteria for diagnosis, can take between 3 to 5 working days to be reported. However, early and appropriate consideration of autoimmune conditions as differentials of an acutely ill patient with multisystem involvement can expedite the process of diagnosis, involvement of related specialty and commencement of appropriate treatment. This approach will benefit patients producing favourable outcomes and minimal hospital stay.

**P44 Table.** Patient’s Laboratory results

Laboratory investigations	Results (admission)	Results (discharge)	Normal value
Haemoglobin	109	106	130-180 g/L
White cell count	21	11	4-11 x 10 ^9/L
Lymphocytes	0.9	1.1	1.0-4.0 x 10^9/l
Neutrophils	19.5	9.9	1.8-7.5 x 10^9/L
Platelet	105	244	150-450 x 10^9/L
ESR	120	39	0-27 mm/hr
CRP	192	34	<10.0 mg/L
Ds-dna antibodies	2.0		<9.9 IU/ml
SS-a antibodies	>8.0		<0.9 AI
SS-a52 antibodies	6.1		<0.9 AI
SS-a60 antibodies	>8.0		<0.9 AI
SS-b antibodies	>8.0		<0.9 AI
RNP 68 antibodies	7.4		<0.9 AI
SM antibodies	>8.0		<0.9 AI
SM RNP	>8.0		<0.9 AI
Ribosomal p	0.2		<0.9 AI
Chromatin	>8.0		<0.9 AI
Jo-1	0.2		<0.9 AI
Scl - 70	0.2		<0.9 AI
Rheumatoid factor	151		0.0-13.0 kU/L
ANCA	Negative		
Anti cardiolipin antibodies	<1.6		0.0-19.9 GPL U/ml
B2 glycoprotein antibodies	<1.5		0.0-19.9 MPL U/ml
Complement C3	0.56	1.10	0.75-1.65 g/L
Complement C4	0.08	0.16	0.14-0.54 g/L
Urea	36	4.3	2.5-7.8 mmol/L
Creatinine	293	84	65-104 umol/L
Sodium	118	138	133-146 mmol/L
Potassium	4.4	3.9	3.5-5.3 mmol/L
EGFR	15	>90	

